# Heterophil-to-lymphocyte ratio varies with habitat fragmentation and canopy cover in a tropical understory insectivore

**DOI:** 10.1007/s10336-025-02321-0

**Published:** 2025-08-27

**Authors:** Vincent Otieno Onyango, Magdalena Ginko, George Gatere Ndiritu, Laurence Cousseau, Janne Heiskanen, Gladys Nyakeru Kung′u, Peter Njoroge, Mwangi Githiru, Petri Pellikka, Luc Lens, Beate Apfelbeck

**Affiliations:** 1 School of Natural Resources and Environmental Studies, Karatina University, P.O. Box 1957-10101, Karatina, Kenya; 2 Department of Environment and Biodiversity, University of Salzburg, 5020 Salzburg, Austria; 3 Zoology Department, National Museums of Kenya, P.O. Box 40658–00100, Nairobi, Kenya; 4 Centre for Research On Ecology, Cognition and Behaviour of Birds, Ghent University, 9000 Ghent, Belgium; 5 Department of Geosciences and Geography, University of Helsinki, 00014 Helsinki, Finland; 6 Finnish Meteorological Institute, P.O. Box 503, 00101 Helsinki, Finland; 7 Wildlife Works, P.O. Box 310-80300, Voi, Kenya; 8 Finnish Southern Africa Cooperation Institute, 10 Schwabe Street, Windhoek, Namibia; 9 State Key Laboratory for Information Engineering in Surveying, Mapping and Remote Sensing, Wuhan University, Wuhan, 430079 China; 10 BirdLife International, Africa, P.O. Box 3502-10100, Nairobi, Kenya

**Keywords:** Forest fragmentation, Habitat quality, Conservation physiology, White blood cells, Leucocytes

## Abstract

Habitat fragmentation and degradation are major problems in tropical forests, leading to a decline in biodiversity. However, it is often unclear which aspects of habitat change are responsible for the decline of specific species. Physiological studies can help identify potential stressors and elucidate causal relationships between habitat change and species decline. In this study, we measured the heterophil-to-lymphocyte (H:L) ratio as an indicator of long-term physiological stress and ecological pressures, such as pathogen exposure or risk of injury, in the tropical understorey insectivore Cabanis’s Greenbul (Placid) (*Phyllastrephus cabanisi placidus*). We then related this ratio to fragment size and forest degradation (canopy cover and vertical vegetation structure). Greenbuls breeding in forest areas with low canopy cover tend to have higher H:L ratios than those breeding in areas with high canopy cover, indicating that low canopy cover creates challenging conditions for greenbuls during breeding. Unexpectedly, however, greenbuls had higher H:L ratios in the largest, most intact forest fragment. These higher H:L ratios in larger fragments may reflect physiological and immunological adjustments to higher ecological pressures, such as predation, parasite prevalence, and competition for territories, due to intact trophic networks. This study therefore advocates concerted efforts to preserve large forest fragments, while smaller fragments should be rehabilitated to strengthen the ecological corridors and networks of these key biodiversity areas, which are of high conservation priority within the isolated mountain peaks of the Taita Hills in Kenya.

**Supplementary Information:**

The online version contains supplementary material available at 10.1007/s10336-025-02321-0.

## Abstract (de)

**Das Verhältnis von Heterophilen zu Lymphozyten variiert mit der Größe von Waldfragmenten und der Baumkronenbedeckung bei einem tropischen Waldvogel.**

Die Fragmentierung und Degradierung tropischer Wälder führt zu einem Rückgang der Artenvielfalt. Oft ist jedoch unklar, welche Aspekte der Lebensraumveränderung für den Rückgang bestimmter Arten verantwortlich sind. Physiologische Studien können helfen, potenzielle Stressfaktoren zu identifizieren und kausale Zusammenhänge zwischen Lebensraumveränderung und Artenrückgang aufzuklären. In dieser Studie haben wir das Verhältnis von Heterophilen zu Lymphozyten (H:L) als Indikator für langfristige physiologische und ökologische Belastungen, wie z. B. die Exposition gegenüber Krankheitserregern oder das Verletzungsrisiko, bei Keniabülbüls (*Phyllastrephus cabanisi placidus*) untersucht und mit der Größe der Waldfragmente und dem Degradierungsgrad (Baumkronenbedeckung und vertikale Vegetationsstruktur) in Beziehung gesetzt. Keniabülbüls sind insektivor und bewohnen das Unterholz tropischer Wälder und sind daher potenziell besonders anfällig gegenüber Lebensraumveränderungen. Unsere Studie zeigt, dass Keniabülbüls, die in Waldgebieten mit geringer Baumkronenbedeckung brüteten, tendenziell höhere H:L-Verhältnisse aufwiesen als solche in Gebieten mit hoher Baumkronenbedeckung. Dies deutet darauf hin, dass eine geringe Baumkronenbedeckung die Brutbedingungen für Keniabülbüls erschwert. Unerwarteterweise wiesen jedoch auch Keniabülbüls, die im größten, intaktesten Waldfragment brüteten, hohe H:L-Verhältnisse auf. Diese erhöhten H:L-Werte in größeren Fragmenten könnten physiologische und immunologische Anpassungen an ökologische Belastungen wie Prädation, Parasiten und Konkurrenz um Reviere aufgrund intakter trophischer Netzwerke widerspiegeln. Diese Studie plädiert daher für den Erhalt großer Waldfragmente und der Restaurierung kleinerer Fragmente, um die ökologischen Netzwerke und Korridore dieser wichtigen Biodiversitätsgebiete zu stärken, deren Erhaltung innerhalb der isolierten Berggipfel der Taita Hills in Kenia eine hohe Priorität hat.

## Introduction

Tropical forests harbour more than half of the world’s terrestrial vertebrate species and are the most species-rich terrestrial biome (Pillay et al. 2021). However, they are under pressure from growing human populations, inefficient agricultural practices, and unsustainable use of natural resources (Gibbs et al. 2010; Laurance et al. 2014). Human activities within and around forests lead to forest fragmentation and simplification of the vegetation structure. This alters the structural composition of forests, as well as the abiotic and biotic conditions, such as vegetation stratification, canopy cover, and microclimatic conditions (Laurance et al. 2002; Renner et al. 2024). These changes threaten all species but especially reduce the survival of specialized ones, such as tropical forest insectivores (Barlow et al. 2016), which may experience reduced availability of critical resources, such as food and nesting sites (Burke and Nol 1998; Zanette et al. 2000; Suorsa et al. 2003). Altered resource availability can have cascading effects on behaviour (e.g. home range sizes and provisioning rates), fitness, and ultimately the distribution and population sizes of forest species (Hinam and Clair 2008; Hinsley et al. 2008; Homyack 2010). While it is evident that habitat change has adverse effects on species communities (Fahrig 2003; Olivier and van Aarde 2017), the underlying processes and mechanisms that determine how populations can adapt to anthropogenic changes at the individual level—through their impacts on physiology and behaviour—remain poorly understood.

Environmental changes can challenge the ability of individuals to obtain sufficient resources from their environment, thus exacerbating trade-offs between growth, reproduction and self-maintenance (Garland et al. 2022). Physiological responses, such as stress physiology and immune function, can indicate how well an organism is coping with environmental change (Cooke et al. 2013) and have been found to be affected by forest degradation (Suorsa et al. 2003; Messina et al. 2018). For example, the concentration of glucocorticoids is commonly used as an indicator of how well an individual is coping with environmental challenges (Romero and Wingfield 2016). However, glucocorticoid levels change rapidly in response to stressors, whereas environmental change causes long-lasting challenges. An alternative method is to examine changes in leucocyte counts (e.g. the Heterophil-to-Lymphocyte [H:L] ratio) resulting from prolonged environmental pressure. These changes are adaptations of the immune system to intrinsic and external conditions and challenges (Davis et al. 2008; Müller et al. 2011; Goessling et al. 2015).

Leukocytes, including heterophils and lymphocytes, play crucial roles in avian immune systems (Campbell 2015). Heterophils are phagocytic cells of the non-specific immune function that circulate during infections, injuries, and inflammations. They are also upregulated in response to different stressors in preparation of potential harmful events (Davis et al. 2008; Campbell 2015). Lymphocytes act within the acquired immune system, such as in response to parasites or viruses (Lemus et al. 2010). The relative proportion of heterophils and lymphocytes in the blood, and thus the H:L ratio, varies with infection status (Pap et al. 2011; Dunn et al. 2013; Clark 2015; Becker et al. 2019), as well as with age, sex (Hõrak et al. 1998; Strehmann et al. 2025), and season (Dariusz et al. 2011; Valdebenito et al. 2021). Additionally, exposure to stressors or corticosterone administration has been linked to an increase in circulating heterophils through the redistribution of leucocytes between organs and blood. This contributes to changes in the leukocyte profile, resulting in an elevated H:L ratio (Dhabhar et al. 1994, 1996; Davis et al. 2008; Müller et al. 2011; Davis and Maney 2018). Thus, stressors can affect both corticosterone levels and H:L ratios; however, the time course differs, with corticosterone rising quickly, while H:L ratios change more slowly but persistently. Therefore, long-term stressors seem to be better reflected by H:L ratios (Davis and Maney 2018). Changes in the leukocyte profile can therefore be a valuable tool for assessing the impact of environmental change on birds.

Studies have shown that the H:L ratio is higher in offspring or adults breeding in low-quality habitat (Johnstone et al. 2012; Maron et al. 2012; Banbura et al. 2013; Ribeiro et al. 2022). For example, forest management practices and forest patch size in northern forests are related to H:L ratios in breeding birds (Krams et al. 2010) and offspring and predict nestling mortality (Suorsa et al. 2004). Habitat loss and fragmentation have long-term detrimental effects on forest birds (Visco et al. 2015). However, more research is needed to understand the full extent of the physiological responses of tropical species to habitat change (Messina et al. 2022). Most studies have focused on the effects of habitat change on temperate birds; however, these results may not fully apply to tropical birds due to their different life histories and evolutionary history in less variable environments (Wiersma et al. 2007; Huey et al. 2012). Therefore, more studies are needed to understand how habitat fragmentation and degradation impact the well-being of birds in tropical forests.

This study investigated the potential impact of forest fragmentation and degradation on the H:L ratio of breeding Cabanis’s Greenbul (Placid) (*Phyllastrephus cabanisi placidus*) (referred to as ‘greenbul’) in the highly fragmented cloud forests of the Taita Hills. We assessed forest fragmentation and degradation as variations in fragment sizes, canopy cover, and vertical vegetation structure. Greenbuls are insectivorous forest specialists that breed in pairs or small family groups in cloud forests in Eastern Africa (Bennun et al. 1996). In the Taita Hills, the size and connectivity of the remaining cloud forest patches continue to decrease (Teucher et al. 2020) and degraded vegetation structure within fragments is associated with reduced food resources (Kung’u et al. 2023). Previous studies on greenbuls have also shown that they have higher corticosterone levels when breeding in small fragments or areas with low canopy cover (Apfelbeck et al. 2024), as well as increased home ranges when canopy cover decreases (Kung'u et al. 2025). However, bacteria killing capacity (a measure of immune function) was not related to forest fragmentation or degradation in adult greenbuls (Sorg et al. unpublished data). As results differed for physiological proxies in earlier greenbul studies and because habitat change likely causes long-term stress and has far-reaching effects on abiotic factors and biotic interactions, we assessed the effects of habitat degradation using H:L ratios. Based on earlier studies, we hypothesized that forest fragmentation and degradation reduce habitat quality for greenbuls during the breeding season when energy demand is high. Therefore, we predicted that greenbuls breeding in areas within small fragments with low canopy cover or degraded vegetation structure would have higher H:L ratios than greenbuls breeding in larger fragments with good canopy cover and vegetation structure. Furthermore, since females invest more than males (i.e. they lay eggs and provide more food to nestlings; Cousseau et al. 2022), we expected them to have higher H:L ratios than males. Additionally, we tested for a relationship between H:L ratios and baseline corticosterone levels to determine whether these two indicators increase together in response to changes in habitat quality.

## Materials and methods

### Study area

**Fig. 1:**
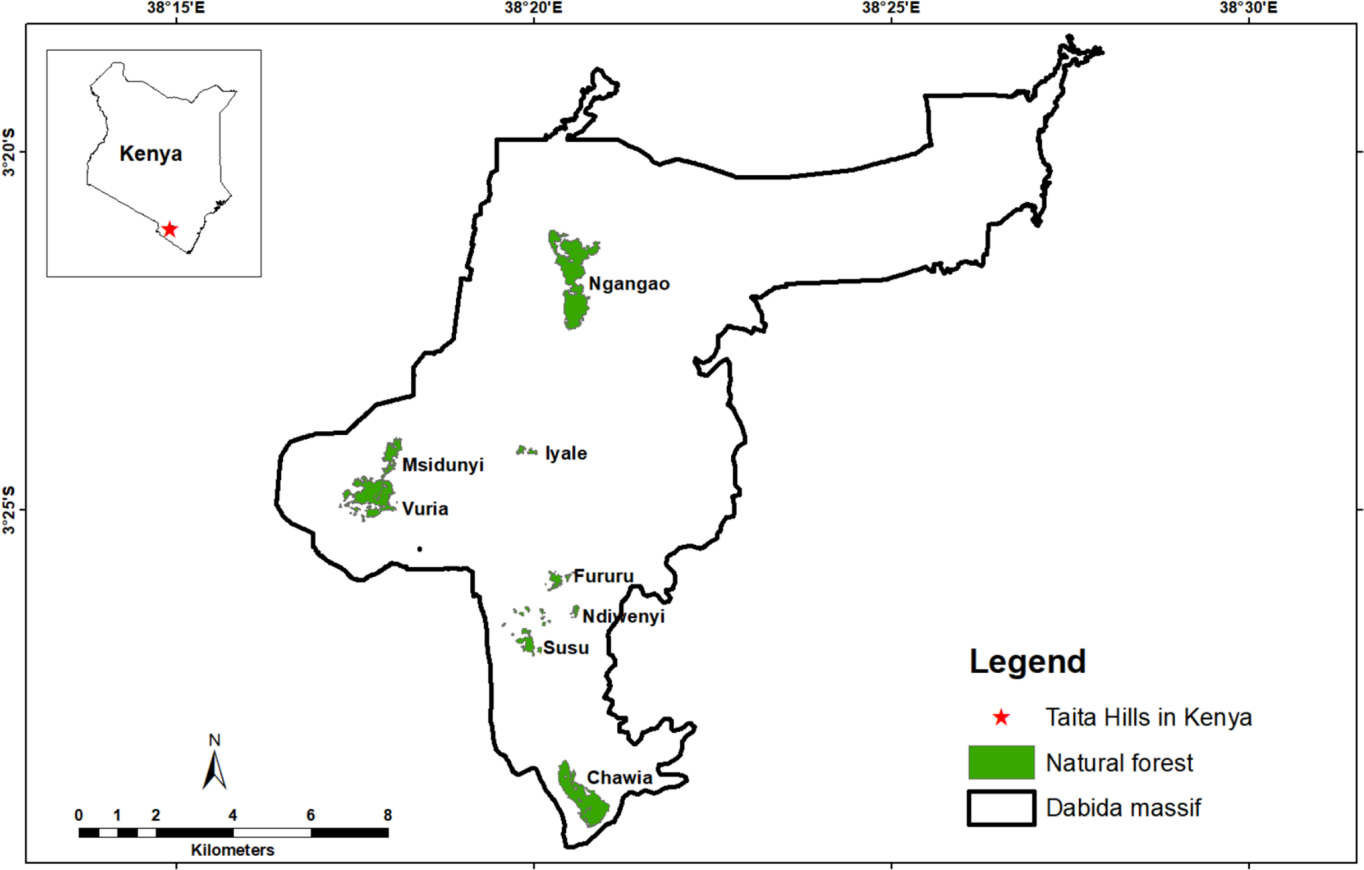
Forest fragments under study in the Taita Hills, Kenya, are indicated in green. These encompass the majority of native forest fragments present in the study area. The boundary surrounding the forest fragments depicts the Dabida Massif

The study was conducted in the Taita Hills in southeastern Kenya, in Taita-Taveta County (3°25'S, 38°20'E; see Fig. 1). The hills are part of the northernmost section of the Eastern Arc Mountains, which are included in the Eastern Afromontane Biodiversity Hotspot (Myers et al. 1999). Due to the high species richness and endemism of their cloud forests, which are under significant anthropogenic pressure, the Taita Hills have been designated one of the world's key biodiversity areas and are a high conservation priority in Kenya (Burgess et al. 2007). Human population growth and the intensification of agriculture, the Taita community's primary economic activity, are major drivers of forest fragmentation. For instance, 260 hectares (50%) of indigenous tropical cloud forest within six fragments were lost to agriculture and bushland in the Taita Hills between 1955 and 2004 (Pellikka et al. 2009). Additionally, weak governance and uncoordinated forest conservation approaches contribute to forest loss and degradation (Teucher et al. 2020). The cloud forests are located in three areas: Sagala Hill, the Mbololo Massif, and the Dabida Massif. This study was conducted in the Dabida Massif, which contains most of the Taita Hills'cloud forest fragments. Specifically, we sampled eight fragments in this massif: two large ones, Ngangao (~130 hectares, near-natural) and Chawia (~86 hectares, degraded); one medium-sized, heavily degraded fragment, Vuria (70.3 hectares); and five small, strongly degraded ones: Ndiwenyi (4 hectares), Fururu (8 hectares), Susu (15 hectares), Iyale (15.7 hectares), and Msidunyi (20.9 hectares) (Fig. 1). Based on weather data obtained from the Kenya Meteorological Department, the Dabida Massif received an average annual precipitation of 1,100 mm between 2010 and 2020. The maximum and minimum temperatures within the same decade averaged 28 °C and 15 °C, respectively.

### Species capture and processing

The study was conducted during three breeding seasons (November to March) in 2016/17, 2018/19 and 2019/20. These seasons varied in weather conditions with the 2019/20 season being wetter and colder than the others (precipitation: 2016/17: 1,000 mm, 2018/19: 983 mm, 2019/20: 1,550 mm; average maximum daily temperature: 2016/17: 29.1 °C, 2018/19: 29.3 °C, 2019/20: 27.6 °C). Greenbul nests within the eight forest fragments were monitored, and individuals were captured in the vicinity of the nests using mist nets (length × height: 3 m × 2.5 m) when nestlings were on average 7 days old (range 5–13 days). Upon capture, a blood sample was taken within 3 min for baseline corticosterone measurements or within 15 min for H:L ratio measurements. Sex was determined by examining the presence of a brood patch (in breeding females) and the size of the cloacal protuberance, which is larger in breeding males than in females. Then, the bird was fitted with a unique combination of three-colour rings and a metal ring. Other biometric measurements recorded included weight, wing length, and tarsus length.

Blood samples were obtained by puncturing the brachial wing vein and collecting blood within heparinized capillary tubes. A drop of blood was put on a microscope slide, spread to a thin smear by pushing a second slide across the first slide and allowed to dry. Absolute ethanol was used to fix the dried blood onto the slide as it has been proven to be as effective as methanol, but is less hazardous (Bhattacharyya et al. 2018). After the field season, slides were stained with a Wright–Giemsa stain following the manufacturer´s instructions (Diff-Quick, LABOR + TECHNIK Eberhard Lehmann GmbH, Germany). Slides were stored in slide boxes in a dry and cool area. Corticosterone levels were obtained over four breeding seasons and have been published elsewhere (see Apfelbeck et al. 2024 for details on laboratory analyses).

### Differential counting of heterophil-to-lymphocyte ratio

Differential counting of white blood cells was performed through examination of the stained thin blood smears using a widefield photomicroscope (Polyvar, Leica) for 71 individuals (66 adult females and five adult males) from 2018/19 and 2019/20 and using a light microscope for 26 individuals (16 adult females and ten adult males) from 2016/17 at a magnification of × 1000 using oil immersion (Dunn et al. 2013). The examination started in an area where the cells were well distributed, typically near the tail end of the blood smear. Counting was then conducted in a snake-like manner (eClinPath.com).

During the examination, the morphology of white blood cells was assessed, and a differential counting was performed. A total of 100 white blood cells per slide were counted, and the numbers of different cell types were recorded. Leucocytes were differentiated into heterophils, monocytes, lymphocytes, eosinophils, and basophils using characteristics such as the shape, size, and staining properties of the nucleus and the presence of granules (Campbell 2015). Each cell type was counted, and to calculate the H:L ratio the number of heterophils was divided by the number of lymphocytes. Samples of different breeding seasons were analysed by two different people. To ascertain similarity between counts, the second person analysing slides from breeding season 2019/20 trained on samples from 2018/19 until values matched previous counts. Training involved eight slides; high similarity between counts was reached after the fourth slide (*r* = 0.99). In addition, four samples from 2019/20 were also analysed twice (*r* = 0.91).

### Environmental variables

Forest degradation affects the availability, stratification, and structure of vegetation. Our previous studies and observations have shown that greenbuls avoid canopy gaps and forage in all layers of the forest, i.e. from ground level to canopy (Kung'u et al. 2025). We therefore quantified vertical vegetation structure (field-based measurement) and canopy cover (LiDAR-based measurement) around nest sites. In addition, we determined fragment sizes, which represent the overall availability of native forest, which may be severely compromised in the smallest fragments.

Vertical vegetation structure around nest sites was assessed in four subplots of 15 m radius, i.e. a central subplot at the nest location and three subplots 50 m away from the nest location (50 m south, 50 m north-east, 50 m north-west) in April and May 2021 and 2022. In each subplot, vertical vegetation was recorded by looking upwards and assessing the presence or absence of vegetation (0/1) within a circle of 0.5 m radius in five height intervals (0–1 m, 1–5 m, 5–9 m, 9–15 m, > 15 m) at five points, resulting in a total of 20 records per sampling plot. We estimated vertical vegetation structure by calculating the Shannon–Wiener diversity index over the five vegetation height intervals and by summing all occurrences of vegetation above 9 m for the 20 sampling points per plot (Bibby et al. 2012). As these were highly correlated (*r* = 0.8, *p* < 0.001), we retained only the latter for further analysis as it showed higher variation. Although these assessments were conducted after the birds were sampled, we do not expect significant changes in the vegetation structure during this time period as the removal of indigenous trees is prohibited in the forest fragments (Kung’u et al. 2023). In addition, we used the sum of occurrences of vegetation above 9 m as an estimate of vegetation structure, which is less affected by the removal or growth of vegetation within the herb or shrub layer.

The percentage of indigenous forest canopy cover at each nest site was calculated within a circle of 0.79 ha using light detection and ranging (LiDAR). LiDAR data were acquired at a mean flight height of 1,450 m aboveground level using an aircraft-mounted Leica ALS60 sensor in January to February 2014 and February 2015 (Adhikari et al. 2017). LiDAR points were classified into ground points and non-ground points, and a digital terrain model (DTM) was computed at 1 m resolution using LAStools software (rapidlasso GmbH). A 3 m height limit was applied to separate ground/understory and canopy returns (Adhikari et al. 2017). Canopy cover was defined as a ratio of the first returns from canopy and all first returns (Heiskanen et al. 2015) and calculated using *lidR* package (Roussel et al. 2020) in R environment (RCoreTeam 2023).

To determine forest fragmentation, fragment sizes were derived from native forest boundary maps of the Taita Hills created from airborne remote sensing images (Pellikka et al. 2009, 2018).

### Statistical analysis

Reference intervals were calculated using nonparametric estimation and the program Reference Value Advisor V 2.1 (Geffré et al. 2011). Reference Value Advisor was also used to detect potential outliers. Statistical analyses were conducted using R version 4.1.2 (RCoreTeam 2023) including the packages *plyr* (Wickham 2011), *dplyr* for data manipulation (Wickham and François 2014), *lme4* for linear models (Bates et al. 2015), and *ggplot2* for graphics (Wickham 2016). Model fit was confirmed by visually assessing normality and homoscedasticity of residuals and by using R package *DHARMa*, which also confirmed that no outliers were present (Hartig 2024).

We tested the relationship between log-transformed H:L ratios and vertical vegetation structure, canopy cover, and fragment sizes in breeding adult greenbuls using linear mixed models. In addition to proxies for habitat quality, we also included sex and sampling year. The sampling year was included to account for potential variation in weather conditions between years or between different observers. As body mass was largely explained by sex, we calculated residual body condition based on body mass and tarsus length for each sex separately. Furthermore, we tested for a relationship between the H:L ratio and baseline corticosterone levels in a separate model, as corticosterone measurements were unavailable for some individuals. Ring number was included as a random effect to account for individuals that had been sampled repeatedly in different years. Continuous predictor variables were standardized to a mean of zero and a standard deviation of one. Based on the variance inflation factor (VIF, *car* package), no multicollinearity issues were detected between the predictor covariates.

## Results

**Fig. 2:**
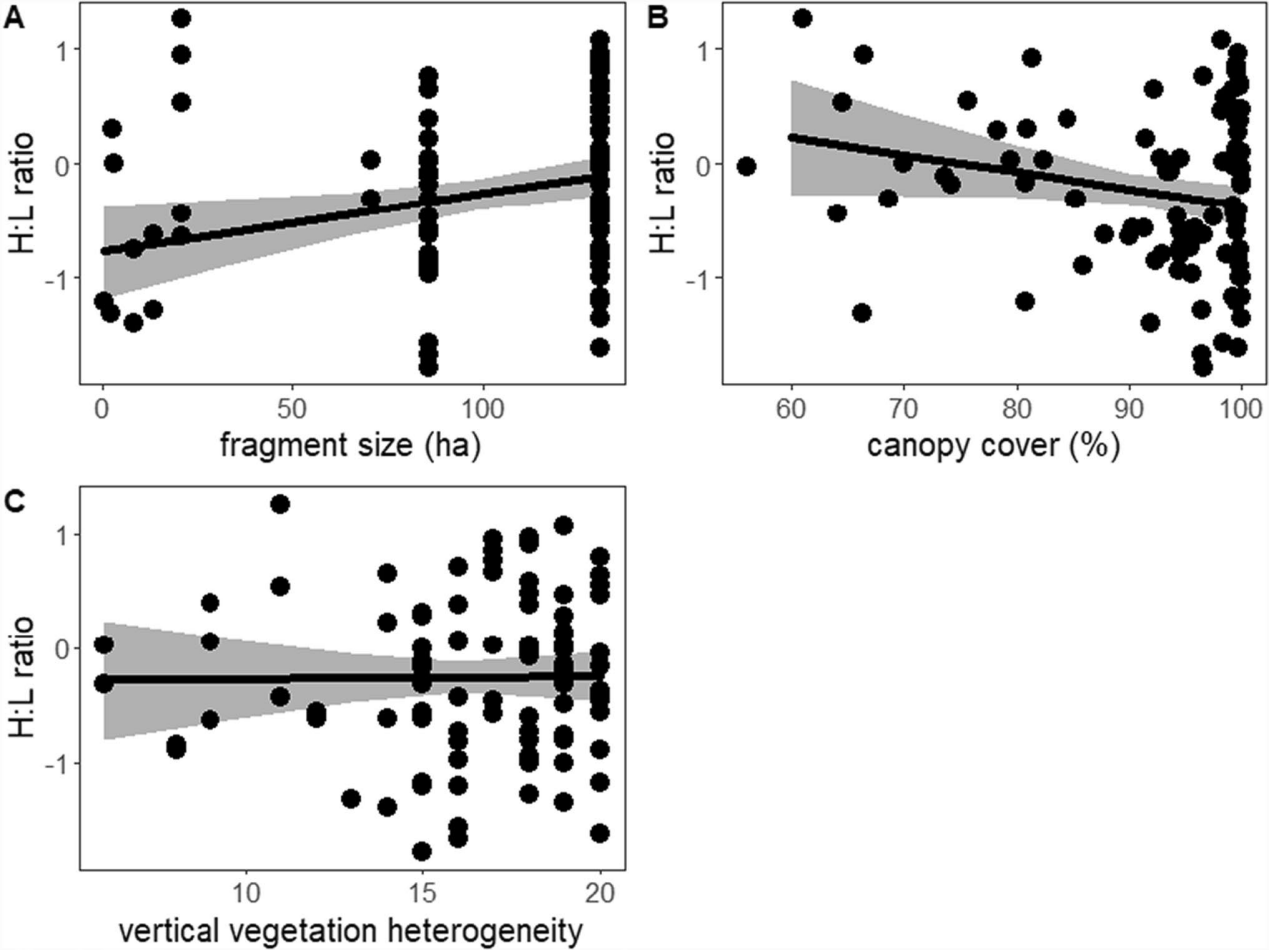
Relationship between H:L ratios and habitat fragmentation and degradation. **A** In larger fragments greenbuls had higher H:L ratios than in smaller fragments. **B** Greenbuls had lower H:L ratios when canopy cover was higher. **C** Vertical vegetation structure was not related to H:L ratio. Note the logarithmic scale for H:L ratio. Shown are raw data points (dots), REML model predictions (line) and 95% confidence intervals (grey shading)

Mean H:L ratio was 0.98 ± 0.68 (range 0.17–3.5). The reference interval for the H:L ratio ranged from 0.2 (90% CI = 0.2–0.3) to 2.8 (90% CI = 2.4–3.5). No outliers were detected, but for the untransformed data three values lay above the upper limit of the reference interval. Greenbuls had higher H:L ratios in larger fragments than in smaller fragments (Table 1 and 2, Fig. 2a). There was also a marginally significant (*p* = 0.05) relationship between H:L ratio and canopy cover with individuals breeding in areas with low canopy cover having higher H:L ratios than those breeding in areas with high canopy cover (Table 2, Fig. 2b). Vertical vegetation structure was not significantly related to H:L ratio (Table 2, Fig. 2c). Males had lower H:L ratios than females and H:L ratios differed between years (Table 2). Residual body condition and baseline corticosterone levels were not significantly related to H:L ratios (corticosterone: *F*_1,75.1_ = 0.05, *p* = 0.83, Table 2).

**Table 1:** Descriptive statistics for the variation in H:L ratios between fragments. Fragments smaller than 21 ha are combined. Additionally, the analysis includes two samples from fragment Vuria, which is intermediate in size but mostly made up of exotic forest, with native forest occupying only a small area

Fragment ID	Mean	Standard deviation	Min	Max	Sample size
Ngangao	1.06	0.67	0.20	2.92	57
Chawia	0.79	0.50	0.17	2.15	25
Small fragments	1.01	0.94	0.25	3.5	15

**Table 2:** Statistics and coefficients for linear mixed-effects models determining the relationship between variation in H:L ratio of greenbuls nesting in areas of varying quality in cloud forest fragments of the Kenyan Taita Hills (97 observations of 81 greenbuls). CI, 95% confidence interval. Significant relationships are indicated in bold

Predictors	Estimates	CI	*df*	*t*-value	*p*-value
Intercept [2016/17; female]	0.19	−0.09–0.48	87.5	1.36	0.18
Year [2018/19]	**−0.38**	−**0.72 – −0.04**	**64.2**	−**2.21**	**0.03**
Year [2019/20]	**−0.60**	−**0.93 – −0.27**	**80.5**	−**3.60**	**0.0005**
Residual body mass	−0.02	−0.11–0.08	84.6	−0.38	0.71
Sex [male]	**−0.54**	−**0.93 – −0.14**	**84.0**	−**2.70**	**0.008**
Fragment size	**0.22**	**0.03–0.41**	**80.5**	**2.30**	**0.02**
Canopy cover	**−0.16**	−**0.33–0.00**	**78.2**	−**1.98**	**0.05**
Vertical vegetation structure	0.003	−0.16–0.16	88.4	0.04	1.0

## Discussion

Contrary to our predictions and previous studies, which have shown that the H:L ratio increases with decreasing habitat quality or size (Suorsa et al. 2004; Krams et al. 2010; Johnstone et al. 2012; Maron et al. 2012; Banbura et al. 2013), H:L ratios in our study were highest in the largest, most intact fragment. In line with our predictions greenbuls breeding in areas with low canopy cover had higher H:L ratios than those breeding in areas with a dense canopy cover.

In a previous study, we found that breeding greenbuls have higher corticosterone levels in areas with low canopy cover (Apfelbeck et al. 2024), which is in line with higher H:L ratios in individuals from areas with low canopy cover. These results indicate that greenbuls encounter more challenging conditions when breeding in areas with reduced canopy cover. A reason may be that greenbuls are reluctant to use canopy gaps and thus have to travel longer distances to foraging patches when canopy cover is low (Kung'u et al. 2025). Thus, increased fragmentation and degradation appears to lead to higher corticosterone levels and H:L ratios, indicating increased workload and energetic needs for nestling provisioning. Consistent with this, breeding females, who contribute the most to nestling provisioning (Cousseau et al. 2022), have higher corticosterone levels (Apfelbeck et al. 2024) and higher H:L ratios (this paper) than breeding males. Across several species, breeding females have been found to have higher physiological indices, including H:L ratios, than males during breeding, which has been interpreted as reflecting the higher parental investment of females in many species (Hõrak et al. 1998; Strehmann et al. 2025). Similar to the previous study on corticosterone levels, vertical vegetation structure was not correlated with H:L ratios. Therefore, adult greenbuls appear to be more impacted by a lack of canopy cover, which forces them to travel greater distances (Kung'u et al. 2025), than by the structure of the vegetation itself. This may indicate flexibility on the part of greenbuls, which we have observed foraging in all vegetation layers, avoiding only areas that completely lack canopy.

The finding of higher H:L ratios in the largest fragment, contrasts with our expectations and also with previous results on corticosterone levels in greenbuls (Apfelbeck et al. 2024). However, some other studies have also found higher H:L ratios in more intact habitats (Mazerolle and Hobson 2002; Kilgas et al. 2006). Mazerolle and Hobson (2002), for example, find that male Ovenbirds (*Seiurus aurocapilla*) from continuous habitat show worse health parameters, including higher H:L ratios, than individuals from fragmented forests attributing it to higher competition for high-quality territories in the more densely occupied contiguous forest. In line with this, territory holders with more unfamiliar neighbours experience greater physiological costs than those with few neighbours in cooperatively breeding Seychelles Warblers (*Acrocephalus sechellensis*) (Bebbington et al. 2017). In our study area, the most intact fragment contains smaller territories, likely due to fewer canopy gaps (Kung'u et al. 2025). Thus, while better quality habitat can support higher population densities (Pérot and Villard 2009), competition for these limited high-quality territories may be increased, which may result in increased physiological costs as reflected by higher H:L ratios.

In contrast, in a previous study we found elevated corticosterone levels in individuals breeding in small fragments (Apfelbeck et al. 2024), which is opposing our findings on H:L ratios and seems to contradict the idea of increased physiological costs due to competition in intact continuous forest. However, although high corticosterone levels, such as stimulated by exogenous hormone treatment, have been shown to lead to an increase in H:L ratios, circulating corticosterone levels of untreated individuals usually do not correlate with H:L ratios (Müller et al. 2011), which was also the case in our study. Indeed, corticosterone levels and H:L ratios may correlate with different environmental stressors (Müller et al. 2011) and may relate to fitness in different ways (Maness et al. 2023). While both respond to acute, severe challenges, albeit with different time-courses, they are affected differently by persistent changes in conditions or stressors. Some researchers have suggested that corticosterone levels primarily reflect coping with short-term challenges and stressors (i.e., days to weeks), while changes in H:L ratios also relate to long-term stressors lasting weeks or months (Goessling et al. 2015; Davis and Maney 2018).

Thus, corticosterone levels may primarily reflect increased effort due to habitat degradation during nestling provisioning, while H:L ratios may also be related to broader environmental differences between the fragments, such as competition for territories, parasites, or predators. Forest fragmentation affects predator communities and thus can affect nest predation rates, although the direction of change may depend on a variety of landscape factors (Tewksbury et al. 2006). For example, some studies in tropical areas found higher nest predation rates in the interior of fragmented habitats or forest reserves (= inverse edge effect; Lahti 2001; Newmark and Stanley 2011; Visco and Sherry 2015) arguing that forest-dependent nest predators favour the interior of (bigger) forest fragments, because of less human disturbance and better habitat quality (Spanhove et al. 2009). However, this makes individuals nesting in the centre, especially in large habitat fragments, more susceptible to nest failures. This is maybe the case in the Taita Hills as well, where nest predation on greenbuls was found to be highest in the centre of the largest, most intact fragment, Ngangao (Spanhove et al. 2014). The presence of predators has been shown to lead to changes in H:L ratio, although the direction of change is not consistent across studies (Tilgar et al. 2010; Caetano et al. 2014; Roncalli et al. 2018).

Similarly, forest fragmentation and degradation affect parasite communities, thereby affecting pathogen exposure (Laurance et al. 2013; Pérez-Rodríguez et al. 2018). Although studies vary in their findings about the relationship between habitat fragmentation and parasite prevalence depending on the host species or parasite considered (Fecchio et al. 2021; Perrin et al. 2023), a study conducted in an Australian tropical rainforest found higher haemosporidian prevalence in continuous forests than in small forest fragments (Laurance et al. 2013). Due to the central role of leucocytes in immune defence, potential changes in parasite abundance and their vectors and thus the probability of infection due to forest fragmentation and degradation, may also explain variation in H:L ratios between fragments (Renner et al. 2016). Thus, higher H:L ratios in larger fragments may reflect an adaptive immune response to the higher risk of injury and pathogen exposure associated with higher levels of competition and predation, rather than poor habitat quality. In our study, whether higher H:L ratios in the larger fragment were related to higher parasite prevalence, increased predation, or competition for territories remains speculative, as we presently lack such information.

In this study. we have focused on breeders, however previous studies in greenbuls and other species have suggested that the effects of habitat fragmentation and degradation may especially affect offspring (Sorg et al. unpublished data, Kung’u et al., unpublished data), which are expected to suffer most directly from resource limitation. For example, Suorsa et al. (2004) found higher H:L ratios in nestlings growing up in small forest patches, but no relationship between forest patch size and H:L ratios in adults. Thus, in future studies nestling H:L ratios should be considered as well. Furthermore, it should be noted that the sample sizes for small fragments and areas with low canopy cover were smaller than those for larger fragments and for areas with high canopy cover. Additionally, samples from the third breeding season were analysed by a different person than those from the first two seasons. While this is not ideal and could lead to variation between breeding seasons, as H:L ratios were higher in the first two breeding seasons than in the third, we have tried to minimize observer bias by training observers and double sampling a subset of slides, for which high interobserver correlations were achieved. Instead, the differences between years may be due to environmental variations, such as weather, as conditions were drier and hotter during the first two breeding seasons than during the third, for which the lowest H:L ratios were found.

## Conclusion

Habitat degradation, particularly the reduction in canopy cover, appears to create challenging conditions for greenbuls during breeding, as reflected in elevated corticosterone levels (Apfelbeck et al. 2024) and H:L ratios. However, higher H:L ratios in the largest, most intact forest fragment suggest that, despite higher potential physiological stress, intact ecosystems may offer greater overall ecological value. These areas likely provide more complete trophic networks, which support higher biodiversity, but may also create stressors such as increased competition for high-quality territories, parasitism, and predation. Our results suggest that habitat fragmentation, while potentially relaxing some stressors (such as reduced competition in smaller fragments), also introduces complex trade-offs. The higher H:L ratios observed in the most intact fragment may reflect the physiological and immunological responses to these multifaceted challenges. These findings, together with previous data on greenbul movement behaviour and physiology (Apfelbeck et al. 2024; Kung’u et al. 2025), highlight the need for conservation strategies that balance both habitat connectivity and quality within forest fragments. Thus, effective conservation efforts in the Taita Hills should focus not only on maintaining connectivity between forest fragments (Githiru et al. 2011), but also on ensuring the preservation of forest quality, which supports the full range of ecological functions and species interactions.

## Supplementary Information

Below is the link to the electronic supplementary material.

Supplementary file1 (XLSX 33 KB)

## Data Availability

All data used in this paper are included in its content and in the electronic supplementary material.
